# Acquired dermal melanocytosis restricted to the hand^⋆^

**DOI:** 10.1016/j.abd.2023.05.013

**Published:** 2024-06-29

**Authors:** Lucas Braga Leite, Flávia Regina Ferreira, Márcia Lanzoni de Alvarenga Lira

**Affiliations:** Service of Dermatology, Hospital Municipal Universitário de Taubaté, Taubaté, SP, Brazil

Dear Editor,

A 48-year-old white female patient presented with greyish-brown macules with a lacy appearance on the palm of her right hand, of 20 years evolution. On physical examination they predominated on the palmar folds at the base of the 3rd, 4th, and 5th fingers (Fig. 1). More recently (2‒3 years) a similar, more discreet lesion appeared on the dorsum of the same hand (Fig. 2). The patient was asymptomatic and there were no triggering factors nor similar lesions on the contralateral side or in any other location. The patient was not menopausal and denied the use of oral contraceptives. There were no comorbidities or family history. An incisional biopsy was performed on the palm, and histopathology revealed a sparse and poorly defined proliferation of spindle cells containing a large amount of melanin, in perivascular and interstitial distribution in the reticular dermis (Figure 3, Figure 4). The cells were immunohistochemically reactive with anti-S-100, HMB-45 and MELAN-A antibodies, consistent with a melanocytic proliferation.

**Figure 1:**
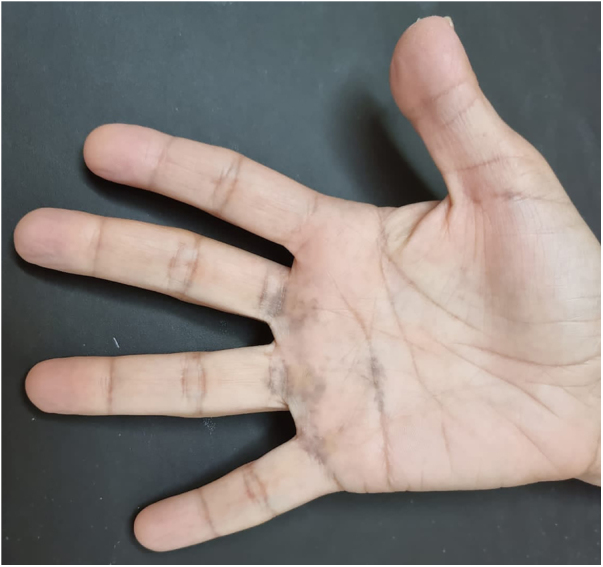
Palmar lines at the base of the 3rd, 4th and 5th fingers: greyish-brown spots with a lacy appearance.

**Figure 2:**
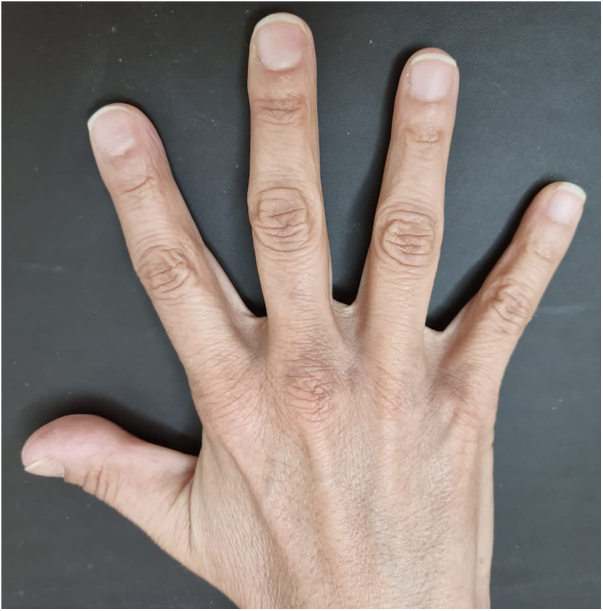
Dorsum of the hand ‒ between the bases of the 3rd, 4th and 5th fingers: grayish-brown spots.

**Figure 3:**
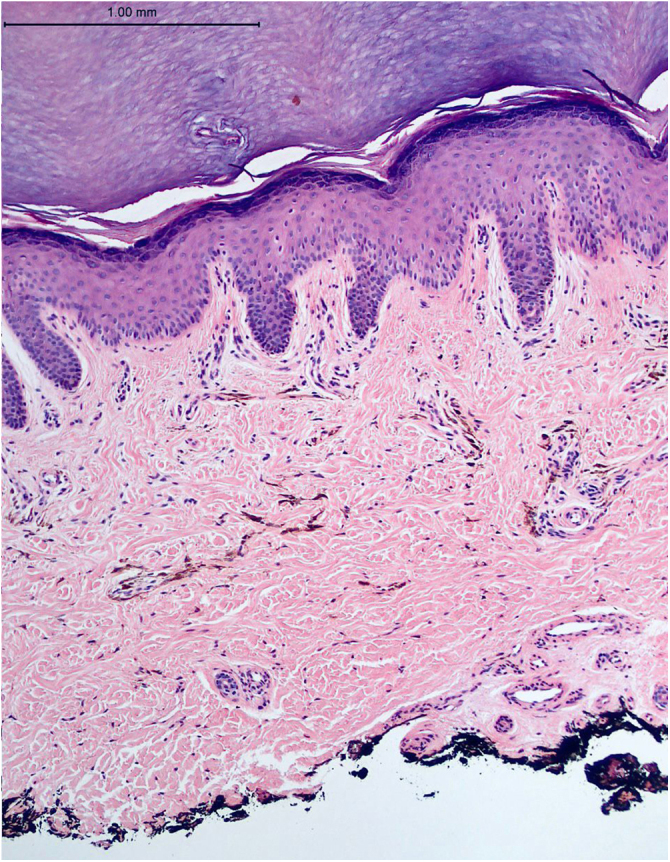
Acral skin ‒ pigmented dendritic cells scattered in the upper reticular dermis. (Hematoxylin & eosin, ×100).

**Figure 4:**
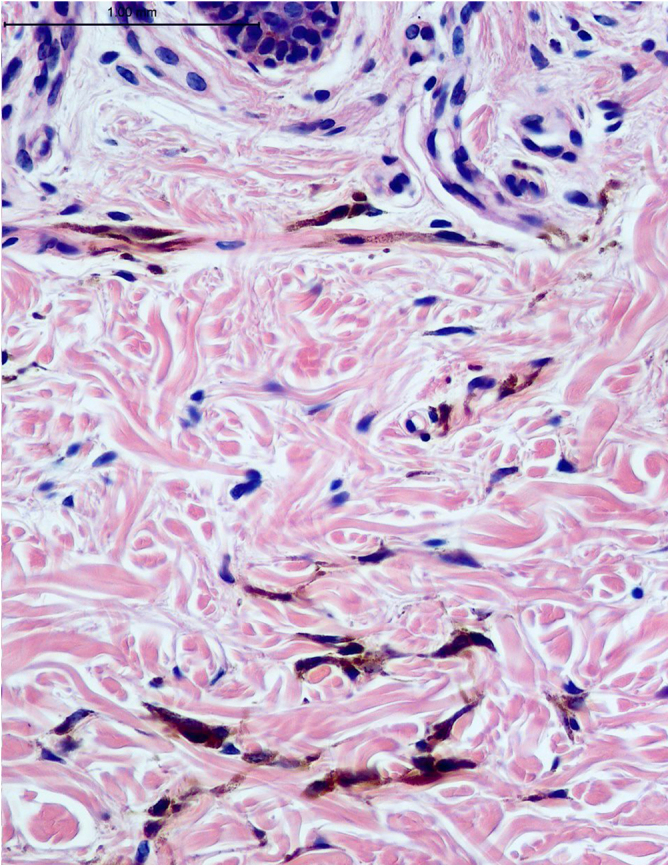
Proliferation of scarce pigmented dendritic cells amidst preserved collagen fibers in the upper reticular dermis (Hematoxylin & eosin, ×400).

Dermal melanocytosis is characterized on histopathology by intradermal melanocytes corresponding to the brownish, grayish and/or bluish spots seen clinically.^1^, ^2^ Dermal melanocytosis can be congenital, such as the Mongolian spot, or can appear after birth or subsequently, such as nevus of Ota, nevus of Ito, and blue nevus. The appearance of lesions in adulthood is extremely rare and scarcely reported. When it does occur, it usually develops in patients with other pre-existing dermal melanocytosis.^3^ Acquired dermal melanocytosis (ADM) was the nomenclature originally proposed by Hori et al. for lesions identified on the face, more commonly in young and middle-aged Japanese women.^4^ Subsequently, lesions with similar characteristics were described in extra-facial topographies, including the trunk and extremities.^2^, ^3^, ^5^ In the present case, the patient showed classic clinical and histopathological features of ADM, restricted to the right hand, of late-onset and without identified triggering factors, in line with the findings of Fukuda et al. and Nakauchi et al.^3^, ^5^ In the literature, ADM of the hands presents relatively smaller lesions when compared to lesions on the face and trunk, and tends to be located on the palmar and juxta-articular lines,^5^ findings that were also detected in this patient.

The etiology of ADM remains unknown but some hypotheses have already been raised; (i) decrease of epidermal melanocytes; (ii) migration of melanocytes from the hair bulb; or (iii) reactivation of pre-existing latent dermal melanocytes due to dermal inflammation, atrophy or degeneration of the epidermis and/or dermis with aging, or other causes.^4^ Estrogens and progestins also seem to play a relevant role in the development of ADM, especially when on the face, corroborated by greater occurrence in young and middle-aged premenopausal women.^1^ In any case, the etiology seems to be multifactorial and its precise determination is likely to be difficult.^6^

On histopathology, melanocytes are cells located in the basal layer of the epidermis, and the diagnosis of dermal melanocytosis is based on the presence of dendritic melanocytes in the dermis.^7^ Melan-A, S-100, and HMB-45 show good specificity for melanocytic lesions and can be used to evaluate melanocyte maturation and are useful in the diagnosis of ADM.^8^ The relationship between ADM and melanoma is rarely discussed in the literature.^9^

Knowledge of ADM, and the possibility of its extra-facial occurrence, contributes to clinical suspicion and subsequent diagnosis of this condition, proving to be extremely important given the possible differential diagnoses that may be established at the time of consultation, including acral lentiginous melanoma, plaque-type blue nevus, ectopic Mongolian spot, and post-inflammatory hyperpigmentation.^10^

The authors encourage new reports of ADM restricted to the hand, consolidating its knowledge by dermatologists, and further studies to elucidate the etiology of this rare condition.

## Financial support

None declared.

## Authors’ contributions

Lucas Braga Leite: Approval of the final version of the manuscript; drafting and editing of the manuscript; collection, analysis and interpretation of data; critical review of the literature; critical review of the manuscript.

Flávia Regina Ferreira: Approval of the final version of the manuscript; drafting and editing of the manuscript; collection, analysis and interpretation of data; effective participation in research orientation; intellectual participation in the propaedeutic and/or therapeutic conduct of the studied cases; critical review of the literature; critical review of the manuscript.

Márcia Lanzoni de Alvarenga Lira: Approval of the final version of the manuscript; drafting and editing of the manuscript; collection, analysis and interpretation of data; critical review of the literature; critical review of the manuscript.

## Conflicts of interest

None declared.
